# Using the Simulated Patient Methodology in the Form of Mystery Calls in Community Pharmacy Practice Research: A Scoping Review Protocol

**DOI:** 10.3390/pharmacy11020047

**Published:** 2023-03-02

**Authors:** Christian Kunow, Bernhard Langer

**Affiliations:** Department of Health, Nursing, Management, University of Applied Sciences Neubrandenburg, Brodaer Straße 2, 17033 Neubrandenburg, Germany

**Keywords:** simulated patient, mystery caller, covert participatory observation, community pharmacies, scoping review

## Abstract

Community pharmacies (CPs) play a major role in health care delivery. The simulated patient methodology (SPM), which is considered the “gold standard”, is recommended for studying CP practice. SPM can be applied in different forms, which include visits and also calls (“mystery calls”). So far, only the role of visits in the investigation of CP practice is known. As the first study worldwide, a systematic map of such reports will be provided, which applies calls in the context of the SPM for the study of CP practice. Reports with the pharmacy staff as the population under study should be included. Reports should be included that conduct an investigation using the SPM in the form of calls to simulate patients and other customers. Reports should be included that examine CP practice as defined by the International Pharmaceutical Federation and the World Health Organization (FIP/WHO). The scoping review methodology will be applied using the associated guidelines from Joanna Briggs Institute (JBI) and PRISMA extension for Scoping Review (PRISMA-ScR). The search will be for both published and unpublished original research in English with transparent information on SPM until the end of 2022. The plan is to search Embase, MEDLINE via PubMed, and Web of Science. Directly afterward, the respective literature collection of the reviewers and the reference lists of suitable international reviews will be searched. This will be followed by a forward and backward snowballing in Google Scholar. For the inclusion of reports, a selection process and for the data extraction a data charting process with the help of variables derived from related reviews and from two SPM-form spanning international guidelines will be performed. The data extracted from the included reports should be synthesized and presented in MS Excel tabular form using the previously determined variables.

## 1. Introduction

Community pharmacies (CPs) and their associated pharmacy staff, as the third largest group of health professionals, play a major role in the health care of their respective populations [1,2]. Moreover, as members of the interprofessional team and key components in individualized patient care, CPs are in a unique position [3,4,5,6,7]. CPs have the advantage of being easily accessible to patients and other customers (who are inquiring about a patient) because of their infrastructure and hours of operation, not requiring an appointment, and providing a more convenient and less formal environment for those who cannot or do not want to use other care facilities [8,9,10,11,12,13]. Thus, CPs enable rapid and preference-based care, especially since CPs are often patients’ first contact with care providers and, in some cases, the only ones providing care to patients [14]. However, the fact that patients and other customers ultimately receive good care depends on the specific way in which CP practice is implemented. The International Pharmaceutical Federation and the World Health Organization (FIP/WHO) define a good CP practice as the “practice of pharmacy that responds to the needs of the people who use the pharmacists’ services to provide optimal, evidence-based care”. FIP/WHO envisage the following four main roles, functions, and activities for CPs: (1) prepare, obtain, store, secure, distribute, administer, dispense, and dispose of medical products, (2) provide effective medication therapy management, (3) maintain and improve professional performance and (4) contribute to improving the effectiveness of the health care system and public health [15].

There are several ways to study CP practice. These include surveys, interviews, clinical vignettes, and non-participatory observations [16,17]. The validity of surveys, interviews, and clinical vignettes is limited due to the social desirability bias since the surveyed or interviewed pharmacy staff generally tend to evaluate their own practice better than it is actually provided in the everyday situation [18]. In the case of non-participatory observations, the disadvantage is that pharmacy staff usually change their behavior (“Hawthorne effect” [19]) when they realize that they are being observed. This behavioral adjustment can lead to practice that is too good compared to the everyday situation. To avoid these problems, the simulated patient methodology (SPM) is recommended [20,21]. SPM is defined in the international literature [22,23,24,25] as a covert participatory observation by a person, ideally indistinguishable from a real customer (simulated patient, SP), who contacts a CP to simulate a real situation based on a defined scenario. Subsequently, data are collected according to previously defined criteria using an assessment form and performance feedback is provided to the CP if necessary. Even though the SPM is associated with a relatively high administrative and financial effort, thereby comparatively small sample sizes [17] and possible intra- and inter-observer variabilities [26], can only investigate a limited number of indications and scenarios [27], and is limited to the investigation of CP practice with customer participation, it is internationally considered the gold standard [17,28,29,30].

SPM can be applied in CP setting in different forms. The most realistic is the visits referred to as “traditional” [31,32], which involve an in-person face-to-face encounter. Although this requires the SP to be physically present in the CP and incurs some effort for observation, with the exception of postal deliveries, this is the only way that the SP can receive the medicines. Another analog application form of SPM is postal letters, which, however, allow a rather impractical observation due to a time-delayed communication and therefore seem to be less suitable for an application. In addition to these two analog applications, a number of digital applications of SPM, which have received a boost in terms of their importance in CP practice from the COVID-19 pandemic [32,33,34,35,36], are also possible such as calls, emails, email chats, and video chats. In contrast to visits, these do not require the physical presence of the SP and thus enable observation with less effort. With emails and email chats, however, as with postal letters, a certain time delay can occur. With video chats, compared to the other digital applications, there is the possibility to observe both verbal and—as with visits—nonverbal communication. However, video chats (still) play a minor role in CP practice [37,38,39,40,41,42,43,44].

In comparison, calls are—right after visits—the most used form of communication between the customers and the CPs [37,38,39,40,41,42,43,44] and are furthermore appreciated by customers [45]. The obvious advantages of calls over visits are that, on the one hand, the customer may feel more comfortable communicating in his familiar private or self-selected environment, where—compared to visits in CP—(lack of) privacy should rather not be a problem. On the other hand, CPs can also be reached by customers who cannot or do not want to visit CPs for a wide variety of reasons (e.g., tied to a place of residence, living remotely in combination with limited access to transportation) [46]. A comparative study of the effect of calls and visits on possible medication-related problems, pharmacy staff interventions, and documentation shows that calls achieved similar—in some criteria even better—results [47]. Even if a visual observation is not possible with calls, they can be a methodological alternative to visits, especially because they are less time-consuming. It is at least proven that calls are the most frequently used communication method for the investigation of digital interventions [31].

It is unclear to what extent calls play a role in the investigation of CP practice. Therefore, a preliminary search for previous reviews in electronic databases was performed using the single search option “title” (Google Scholar via Google interface) or “title/abstract” (MEDLINE via PubMed via NCBI interface). Two terms (“review” AND “pharmacy” OR “pharmacies” OR “pharmacist” OR “pharmacists”) were used in combination (AND) with the term “simulated patient” most commonly used in SPM reports in the form of visits [24] or (OR) the term most obvious here (“simulated call” OR “simulated calls” OR “simulated caller” OR “simulated callers”) or (OR) the term already known to the authors (“mystery call” OR “mystery calls” OR “mystery caller” OR “mystery callers”) [48]. It could be determined that both internationally [22,23,24,49] and nationally [50] the SPM, albeit in the form of visits, has been frequently used to investigate CP practice. An older, current, or ongoing study on mystery calls does not seem to exist yet.

## 2. Review Question

In addition, against the background that reviews of applied methods in health care or in specific settings are not uncommon [51,52,53,54], the objective is to be the first study worldwide—in particular, to differentiate it from visits—to provide an insight into the application of SPM in the form of calls in CP practice research based on a variety of application aspects. For this purpose, a systematic map and summary of relevant reports will be provided, thus capturing the extent of available evidence on a rather broad topic and identifying possible knowledge gaps. The following key research question is formulated: What is known about the use of SPM in the form of mystery calls to investigate CP practices? In order to answer this question and achieve the above-mentioned objective, the appropriate scoping review methodology will be applied.

The scoping review protocol reported here was developed to plan and conduct an appropriate scoping review. The guidelines of the Joanna Briggs Institute (JBI) [55] and the JBI Scoping Review Methodology Group [56] were used for development and reporting. Current scoping review protocols [57,58,59] that do not include sections on discussion, limitations, and conclusions, as also provided in the guidelines, serve as practical examples.

## 3. Eligibility Criteria

The prefixed title of this scoping review protocol and that of the subsequently planned scoping review with a nearly identical title, the previously posed review question, the following inclusion criteria, and the subsequent search strategy are guided by the population, concept, and context (PCC) framework.

### 3.1. Population

Reports with the pharmacy staff as the population under study should be included. Pharmacy staff includes pharmacists or non-pharmacists (other health professionals), and persons in vocational training/study or with a professional/academic degree regardless of their characteristics (e.g., age, gender, or ethnicity). The pharmacy staff must work for a CP. Conversely, reports with pharmacy staff in other pharmacy settings (e.g., online pharmacies, hospital pharmacies) or non-pharmacy settings (e.g., hospitals) should be excluded.

### 3.2. Concept

Reports should be included that conduct an investigation using SPM in the form of mystery calls. Conversely, reports should be excluded that use a different form of SPM, e.g., visits, or a different methodology such as surveys, interviews, clinical vignettes, or non-participatory observations. In the sense of the definition of SPM (see Section 1), only reports that simulate customers (patients and persons who are inquiring for a patient) should be included. Conversely, reports that simulate other groups of people (e.g., government employees) should be excluded.

### 3.3. Context

Reports investigating the practice of CPs should be included. The CP practice should be the main roles, functions, and activities foreseen by the FIP/WHO for CPs, thus excluding reports that do not investigate a CP practice (e.g., interior design).

### 3.4. Types of Evidence Sources

In order to be able to present as comprehensive an overview as possible and a wide variety of evidence as well as to show trends or changes with regard to the use of mystery calls, there is no specific period of investigation. Thus, reports should be included by the end of 2022. In order to cover the totality of available evidence on a rather broad topic, the inclusion should be independent of the country of the study site, the investigated medicines or indications, and the study design of the reports (qualitative, quantitative, mixed methods) and also independent of whether reports report on the same investigation. Due to the subject of the study, the reports must be original research with transparent information about the SPM, which excludes pure abstracts and reviews. However, reports that have been investigated in reviews and that meet the eligibility criteria will be included. To minimize publication bias, both published reports (white literature) and unpublished reports (grey literature) will be included, regardless of whether they have been peer-reviewed. Due to language barriers, feasibility, and limited resources for professional translation services, the main text of the reports must be written in English to be included.

## 4. Methods

### 4.1. Overview

The guidelines developed by JBI [55] will be used to conduct the scoping review planned here, and the guidelines of the “Preferred Reporting Items for Systematic Reviews and Meta-Analyses (PRISMA) extension for Scoping Reviews (PRISMA-ScR)” [60], which are consistent with the JBI guidelines, will be used for reporting.

First, the objective, methods, and reporting of the planned scoping review were defined in advance, documented on the basis of the present scoping review protocol, and thus made transparent and publicly available. Thus, according to the JBI guidelines, registration is not required [55]. Ideally, the planning in this scoping review protocol should not deviate from the subsequent implementation of the scoping review.

The following methodological steps were planned by two reviewers (CK and BL) who have previously published two reviews, one review being thematically related to the present one [50,61]. One reviewer (CK) should perform all steps with prior pilot testing and the other reviewer (BL) should spot-check the results for plausibility, completeness, and accuracy. Discrepancies should be discussed between both reviewers and clarified by consensus.

### 4.2. Search Strategy

Initially, a search in electronic databases is planned. The following databases will be used at reasonable or no cost: Embase (Ovid interface), MEDLINE via PubMed (NCBI interface), and Web of Science (Clarivate Analytics interface). In Table 1**,** the search strategy is presented as an example for MEDLINE via PubMed. Both standardized terms from the Medical Subject Headings (MeSH) vocabulary and non-standardized terms should be used to broaden the search. The standardized terms were determined manually from the homepage of the National Library of Medicine [62]. The non-standardized terms were identified via various sources. Thus, the selection of search terms with respect to the practice of pharmacy staff in CPs is based on the results of a suitable international review [24]. Thus, from the titles of the 148 papers included therein, the respective terms used for this purpose were extracted as search terms. Even if a corresponding term could be found in more than 80% of the titles, “title/abstract” should be searched for if possible. Regarding the selection of search terms with respect to mystery calls, no synonyms were known before. In order to be able to search as broadly as possible, synonyms of the rather general term “calls” as well as synonyms of the special term “mystery” should be used individually. The search terms of the special term “mystery” are based on the search terms used in international reviews [22,23,24,49]. Additionally, in light of the fact that only less than 40% of the titles of the 148 included papers of the previously mentioned review [24] contained a corresponding term, the search should also be conducted in “title/abstract”. The search terms of the general term “calls” are based on synonyms found by searching the Collins online dictionary [63], in doing so the search should also be performed in “title/abstract”. Besides the search for English language reports until the end of 2022, no other search limitations in the electronic databases are planned. Directly after the search in the electronic databases, the respective literature collection of the two reviewers (BL and CK) and the reference lists of suitable international reviews [22,23,24,49] will be searched. The reports identified at the end should additionally be entered into Google Scholar and searched there for suitable reports, if necessary, as grey literature, which cites the identified reports (forward snowballing) [64]. Afterward, the references of the identified reports should be searched for further suitable reports (backward snowballing) [64]. These two additional search processes should be performed in a recurring cycle until no more suitable reports can be identified. Due to a lack of human resources, there are no plans to contact the authors of the identified reports with requests to provide potentially relevant literature. As recommended in the guidelines [56] searching can be modified and expanded as needed during the review process. These changes—if they occur—will be described in the final scoping review.

### 4.3. Source of Evidence Selection

Afterward, a selection process with the help of the inclusion criteria mentioned before should be accomplished. The corresponding results should be recorded in a selection form, which was developed due to quite a special process. The word file of this form is available from the corresponding author upon reasonable request. With respect to the identified records via databases, first, the duplicates should be removed. Then, the titles and, if available, the abstracts of the records are screened, and the potentially irrelevant records are excluded (level 1). The full texts of the remaining potentially relevant reports should be screened together with the full texts of the identified potentially relevant reports via other methods (literature collection and reference lists) and assessed for eligibility (level 2). The identified non-relevant reports should be excluded, giving reasons. Conversely, the relevant reports identified together with the relevant reports identified by forward and backward snowballing should result in the included reports. The entire process should then be mapped in a flow diagram derived from the PRISMA-ScR [60].

### 4.4. Data Extraction

After the self-development of a permanently refined data charting form (Table 2) for the determination of variables, the data extraction from the included reports should be performed with its help. For this, the main file of the reports and possible appendices/supplements should be used. At first, the data extraction will be performed using basic variables, which were derived from the variables of the PICOS tool [65]. Afterward, the data will be extracted using a few major variables and numerous minor variables that are targeted to the objective and research question at hand. These were mainly derived from variables used in related reviews [22,23,24,49,50,66,67] or found in the SPM-form spanning guidelines for health care simulation research [68] and “checklist for reporting research using simulated patient methodology” (CRiSP) [69].

### 4.5. Data Analysis and Presentation

Based on the present objective and research question, the data extracted from the included reports should be synthesized and presented in MS Excel tabular form using the previously determined variables. Identified trends or patterns should be reported in the results. Due to the heterogeneity of the data, no summary measures, e.g., a meta-analysis, should be performed, whereby this is not foreseen in the guidelines of JBI and PRISMA-ScR [55,60].

## 5. Appraisal of Evidence

In order to focus on the objective and research question at hand, a critical appraisal (methodological quality or risk of bias) of the source of evidence, which is typically performed using an appraisal tool, should be avoided. According to the guidelines, this is only optional and would also contradict the general aim of a scoping review to cover the extent of the available evidence on a rather broad topic [55,60]. Nevertheless, an appraisal of evidence should take place in a certain way, according to which the reporting quality of the included reports can be shown by the examined SPM variables.

## 6. Ethics and Dissemination

There was no involvement of patients or members of the public in this scoping review protocol. Neither patients nor members of the public will be involved in the planned scoping review. Only secondary data will be collected by extraction from included reports. Because of this, ethics approval from an Institutional Review Board was not and is not required. The general dissemination strategy includes that the planned scoping review will be published in a peer-reviewed journal as open access. In addition, it is planned to send the published scoping review to the corresponding authors of included reports via email and thus disseminate it in a very targeted manner. In this way, they can initiate a possible optimization in the application and reporting of future SPM studies in the form of mystery calls.

## 7. Scoping Review Timeline

In October 2022, a preliminary search was performed. It is expected that a piloting search in MEDLINE via PubMed, a piloting selection process, and a piloting data charting process will take place in mid-2023, after the publication of this scoping review protocol. In case of a successful pilot testing with a possible adaptation of the search strategy, the selection form, and the data charting form, the final search, selection process, and data charting process is planned for late 2023. Analysis, presentation, and interpretation of the evidence are scheduled to occur immediately thereafter, so the anticipated date for full preparation of the final scoping report is early 2024.

## Figures and Tables

**Table 1:** Draft of the search strategy for MEDLINE via PubMed.

**Search Terms**		**Term Number**
(1)	pharmacy staff (tiab) OR pharmacy team (tiab) OR pharmacists (MeSH Terms) OR pharmacies (MeSH Terms) OR pharmacy (MeSH Terms) OR store* (tiab) OR “drug store*” (tiab) OR drugstore* (tiab) OR “medical store*” (tiab) OR retail* (tiab) OR sell* (tiab) OR provider* (tiab) OR facilit* (tiab) OR outlet* (tiab) OR shop* (tiab) OR “pharmacy research” (MeSH Terms) OR “evidence-based pharmacy practice” (MeSH Terms) OR “community pharmacy services” (MeSH Terms) OR “pharmaceutical services” (MeSH Terms)	19
(2)	“patient simulation” (MeSH Terms) OR simulat* (tiab) OR standardi* (tiab) OR undercover (tiab) OR myster* (tiab) OR secret* (tiab) OR pseudo* (tiab) OR cover* (tiab) OR surroga* (tiab) OR disguis* (tiab) OR fictitious* (tiab) OR fals* (tiab) OR pose* (tiab) OR posi* (tiab) OR unidentifi* (tiab) OR fake* (tiab) OR confederat* (tiab) OR anonymous* (tiab)	18
(3)	telephone (MeSH Terms) OR smartphone (MeSH Terms) OR “cell phone” (MeSH Terms) OR “cell phone use” (MeSH Terms) OR call* (tiab) OR ring* (tiab) OR buzz* (tiab) OR tinkle* (tiab) OR bell* (tiab)	9
**Search**	**Search Algorithm**	**Search Combinations**
## 1–3.078	(1) AND (2) AND (3)	3.078 (19 × 18 × 9)
Limit for ## 1–3.078: English language and until the end of 2022		

**Table 2:** Data extraction instrument.

Variable	Categories
**1. Basic report information**	
Lead author	Last name OR n/s
Corresponding author	Last name OR n/s
Year of report (first e.g., epub)	Year OR n/s
Title of report	Title OR n/s
Medium of report	E.g., journal name
DOI of report	DOI OR n/s
Pilot report (pilot study)	Yes OR no OR n/s
Follow-up report (follow-up study)	Yes OR no OR n/s
Execution of report (data collection)	Year OR n/s
Location of report	E.g., country, city, region OR n/s
Ethical approval	Yes OR no OR n/s
Report design	E.g., CS OR n/s
Purpose of report (CP practice)	E.g., counseling
Purpose of report (indication/medication)	E.g., headache/analgesic OR n/a
Funding of report	Yes OR no OR n/s
Conflict of interest of authors	Yes OR no OR n/s
Medicinal product market	POM OR OTC OR n/s
**2 Use of the SPM in the form of calls—General**	
**2.1 Terms, definitions and guidelines**	
Term in title	E.g., mystery calls OR n/s
Term in abstract	E.g., mystery calls OR n/s OR n/a
Term in keywords	E.g., mystery calls OR n/s OR n/a
Definition of the SPM	Definition OR n/s
SPM guidelines (e.g., for reporting)	E.g., CRiSP OR no OR n/s
**3 Use of the SPM in the form of calls—Specific**	
**3.1 Scenarios**	
Basis of the development of scenarios	E.g., guidelines OR n/s
Number of scenarios	Number OR n/s
Type of scenarios	E.g., medication-based OR n/s OR n/a
Affected person/requirement	Caller OR third party OR n/a
Requirements for answers	Yes OR no OR n/s
Materials used in the scenarios (e.g., informational)	Yes OR no OR n/s
Validation by pretests (pilot report)	Yes OR no OR n/s
Validation by other measures	E.g., SPM-expert OR pharmacist OR n/s
Change to the planned scenarios	Yes OR no OR n/s
**3.2 CPs**	
Number of CPs	Number OR n/s
Kind of finding of CPs	E.g., list of provider OR n/s
Sample size of CPs (absolutely)	E.g., 120 of 120 OR n/s
Sample size of CPs (percentage)	E.g., 100% OR n/s
Sampling type of CPs	E.g., random OR n/s
Advance information of CPs	Yes OR no OR n/s OR n/a
Subsequent information of CPs	Yes OR no OR n/s OR n/a
Option out for CPs	Yes OR no OR n/s OR n/a
Time between advance information and calls of CPs	E.g., 5 weeks OR n/s OR n/a
Time between subsequent information and calls of CPs	E.g., 5 weeks OR n/s OR n/a
Informed consent from CPs	Yes OR no OR n/s
**3.3 Callers**	
Number of callers	Number OR n/s
Training of callers (theory)	Yes OR no OR n/s
Training of callers (practice)	Yes OR no OR n/s
Age of callers (per caller, mean, median)	E.g., 19 (male), 20 (female) OR n/s
Gender of callers	E.g., 2 males, 3 females OR n/s
Social background of callers	E.g., students OR n/s
Behavior characteristics of callers (nonverbal)	E.g., heavy breathing OR n/s
Detection of callers	Not assessed OR assessed OR n/s
Conflict of interest of callers	Yes OR no OR n/s
Informed consent from callers	Yes OR no OR n/s
**3.4 Calls**	
Type of calls	Formal OR informal OR n/s
Number of calls planned	Number OR n/s
Number of calls completed	Number OR n/s
Number of incomplete calls	Number OR n/s
Reasons for incomplete calls	E.g., detection of the caller OR n/s
Number of planned calls that have been replaced	Number OR n/s
Call completion rate	E.g., 100% OR n/s
Time length of calls (e.g., mean, median)	E.g., 2.15 min. (mean) OR n/s
Number of calls per scenario	Number OR n/s
Number of calls per CP	Number OR n/s
Number of calls per caller	Number OR n/s
**3.5 Assessment forms and items**	
Basis of the development of the assessment form	E.g., guidelines OR n/s
Number of items	Number OR n/s
Kind of items	E.g., dichotomous OR n/s
Validation by pretests (pilot report)	Yes OR no OR n/s
Validation by other measures	E.g., SPM expert OR pharmacist OR n/s
Change to the planned assessment form	Yes OR no OR n/s
**3.6 Data collections**	
Type of data collection (assessment form)	E.g., paper-based OR digital OR n/s
Time of data collection (assessment form)	E.g., right after calls OR n/s
Use of influencing factors	Yes OR no OR n/s
Number of influencing factors	Number OR n/s
Audiotaping for the data collection	Yes OR no OR n/s
Second observer for the data collection	Yes OR no OR n/s
**3.7 Performance feedbacks**	
Form of performance feedback	E.g., personally OR n/s
Individual performance feedback right after call	Yes OR no OR n/s
Individual performance feedback after last call	Yes OR no OR n/s
General performance feedback after last call	Yes OR no OR n/s

n/s = not specified, n/a = not applicable, CS = cross-sectional, POM = prescription only medicine, OTC = over-the-counter medicine.

## Data Availability

Not applicable.
